# ADAPTING THE MEANING OF HOME QUESTIONNAIRE FOR PEOPLE WITH DEMENTIA

**DOI:** 10.1093/geroni/igae098.0539

**Published:** 2024-12-31

**Authors:** Janina Florack, Susanne Penger, Julia Haberstroh, Frank Oswald

**Affiliations:** University of Siegen, Siegen, Nordrhein-Westfalen, Germany; Psychological Aging Research, University of Siegen, Siegen, Nordrhein-Westfalen, Germany; Psychological Aging Research, University of Siegen, Siegen, Nordrhein-Westfalen, Germany; Goethe University Frankfurt, Frankfurt am Main, Hessen, Germany

## Abstract

Most people with dementia prefer to remain in their homes for as long as possible, despite facing various challenges in daily life due to cognitive decline. They generally decide against relocating to a nursing home, underscoring the significant value they attribute to their own residence. So far, the subjective meaning of home in people with dementia has primarily been explored through qualitative approaches. Thus, the objective of the current study was the adaption of an existing questionnaire for assessing the meaning of home (MoH) in people with dementia. In a pilot study, the MoH was revised by simplifying existing items and instructions and adding new items. Testing of the modified version (MoH-D) in a small sample of cognitively impaired individuals (N = 30) necessitated further adjustments, such as simplification of the response scale. In a second study, the factorial structure of the MoH-D was explored in a cognitively unimpaired sample (N = 574), resulting in a preliminary 21-item version with a five-factor structure that demonstrated psychometrically satisfactory characteristics. These factors were labeled comfort, everydayness, sociability, overwhelm, and autonomy based on their content. To further validate and refine the questionnaire, a sizable sample of cognitively impaired people (N = 282) was recruited. Currently, analyses are in progress to determine the final factorial structure of the MoH-D, evaluate its psychometric properties, and investigate its relations with external validation criteria (e.g., housing satisfaction, duration of residence). Initial findings will be presented and discussed.

